# Dietary Fibers in Healthy Children and in Pediatric Gastrointestinal Disorders: A Practical Guide

**DOI:** 10.3390/nu15092208

**Published:** 2023-05-06

**Authors:** Silvia Salvatore, Maria Serena Battigaglia, Elena Murone, Eugenia Dozio, Licia Pensabene, Massimo Agosti

**Affiliations:** 1 Pediatric Department, Hospital “F. Del Ponte”, Via F. Del Ponte 19, University of Insubria, 21100 Varese, Italy; massimo.agosti@uninsubria.it; 2 Department of Medical and Surgical Sciences, Pediatric Unit, University Magna Graecia of Catanzaro, Viale Europa, Germaneto, 88100 Catanzaro, Italy; mariaserena.battigaglia@studenti.unicz.it (M.S.B.); elenamurone@gmail.com (E.M.); pensabene@unicz.it (L.P.); 3 Dipartimento di Medicina e Chirurgia, University of Insubria, 21100 Varese, Italy; eugenia.dozio@uninsubria.it

**Keywords:** dietary fiber, children, prebiotics, fermentation, microbiota, SCFAs, functional gastrointestinal disorders, inflammatory bowel disease, diet

## Abstract

Dietary fibers include non-digestible plant carbohydrates, lignin and resistant starch. Dietary fibers provide immune, cardiovascular, metabolic and intestinal beneficial effects in humans. Fibers naturally present in foods (fruits, vegetables, legumes, cereals) or used as supplements have different physical, chemical and functional profiles. This narrative review provides an update to the knowledge on the effects of dietary fibers in healthy subjects and in children with gastrointestinal disorders. Soluble fibers are digested by gut bacteria, producing short-chain fatty acids and energy for colonocytes, and may exert prebiotic effects that promote the growth of bifidobacteria and lactobacilli. Non-soluble fibers are bulking agents and may improve intestinal transit. The exact amount and characteristics of the fiber requirement in infants and children need to be further established. There are limited data evaluating fibers in children with gastrointestinal disorders. The low intake of fibers has been associated with constipation, but the intake of excessive fibers is not recommended as it may cause flatulence and abdominal discomfort. Certain fibers (particularly psyllium in irritable bowel syndrome) have shown beneficial effects in children with gastrointestinal disorders, but the limited and heterogenous data do not currently allow a specific recommendation.

## 1. Introduction

Fiber is an important dietary component that has gastrointestinal, cardiovascular, metabolic and immune health effects [1,2,3,4,5,6]. Dennis Burkitt was the first physician to suggest that a low intake of dietary fibers could be related to human intestinal tumors [7]. It was later recognized that fibers modulate cholesterol and glycemic levels, gastric emptying, intestinal movement, gut microbiota and the production of short-chain fatty acids (SCFAs) [6]. However, the optimal intake and type of fiber that can improv gastrointestinal wellness are not yet well known [2,6]. 

The aim of this narrative review is to provide clinicians with an update on the characteristics of dietary fibers and their effect on gastrointestinal disorders in children. 

## 2. Definition and Type of Fibers

Two famous ancient Greek doctors, Hippocrates and Galenus, living in the fifth and second centuries BC, first noticed that some foods, such as coarse wheat and brown bread, had beneficial intestinal effects in humans [2]. Two thousand years later, Kellogg reported the laxative and health properties of whole grain [2]. In 1953, the term “dietary fiber”, referring to the non-digestible constituents of the plant cell wall, was introduced [8]. Subsequently, this definition has been expanded and modified several times [9,10]. Dietary fibers include a heterogeneous group of substances: the plant non-starch polysaccharides (e.g., cellulose, pectin, gums, hemicelluloses, β--glucans, and components of oat and wheat bran), plant carbohydrates that are not recovered by alcohol precipitation (e.g., inulin, oligosaccharides, and fructans), lignin, chitin, and some resistant starch [1,10]. Natural fibers refer to dietary fibers that are intrinsically part of the cell wall material or the inner layer of edible plants, fruits, vegetables, cereals, nuts, pulses, and seaweed [5]. In addition, fibers may be extracted from plant or food and modified by different processing methods [5].

The Codex Alimentarius considers dietary fibers to be carbohydrate polymers with ten or more monomeric units, and those that are not hydrolyzed by the small intestinal enzymes in humans. It also identifies three different categories: fibers that are intrinsic and intact; fibers extracted from foods by physical, chemical, or mechanical methods; and fibers that are synthesized or modified. It is noteworthy that fibers in the last two categories require scientific proof of physiological benefits [9]. According to the International Carbohydrate Quality Consortium, dietary fiber is a generic term describing non-absorbed plant wall carbohydrates and non-carbohydrate components such as lignin, and some intracellular storage oligosaccharides, such as fructans [5]. Despite slightly different existing definitions, dietary fibers are recognized to be a group of carbohydrate polymers and oligomers that cannot be digested in the human small intestine and pass into the large bowel, where they can be not, partially or completely fermented by the gut microbiota [9]. In the colon, fibers provide energy fuel and, after metabolization, short-chain fatty acids (SCFA), decrease the pH (improving absorption of certain minerals and contrasting selected pathogens), stimulate the growth of lactobacilli and bifidobacteria, or, if not fermented, act as a bulking agent and stimulate intestinal motility [11,12]. Functional fibers refer to fibers that have a proven beneficial effect in humans [13]. Certain fibers with a carbohydrate structure (e.g., inulin, fructooligosaccharides and galactooligosaccharides) also act as prebiotics [5], being “a substrate selectively utilized by host microorganisms conferring a health benefit” [14]. Prebiotics are functional food components that can be naturally present in plant-based foods or are synthetically produced via the enzymatic conversion of sugars [12]. 

**Figure 1:**
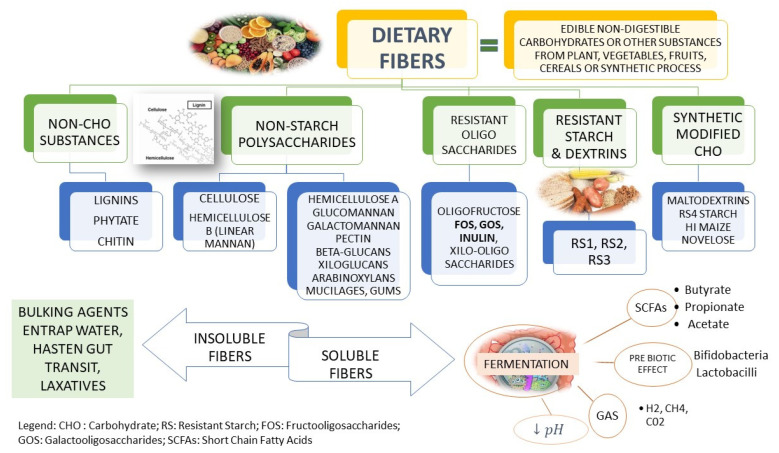
Type and characteristics of dietary fibers.

Dietary fibers present distinct chemical, physical and functional properties [5] (Figure 1). 

Dietary fibers can be differentiated based on their source (e.g., plant cell walls, intracellular components, outer layer of cereal seeds), origin (fruits, vegetables, legumes, grains, synthetic process), degree of polymerization, viscosity, gelification, solubility, and fermentability. Plant cell walls contain different type of fibers, including cellulose, hemicellulose, pectin and lignin, whilst mucilages and gums are in the inner part of the plant. All foods of vegetable origin provide fibers, with a variable content of soluble and non-soluble fractions [10,15]. Table 1 reports the fiber total and soluble content in common foods, fruits, vegetables, nuts, legumes and grains [10,16].

Soluble fibers dissolve in aqueous media in the laboratory [5]. Selected water-soluble fibers have beneficial metabolic effects, such as lowering the blood cholesterol and postprandial glycaemia; this is by increasing the blood’s viscosity and slowing down gastric emptying and the digestion of starch and other macronutrients. The viscosity property of a soluble fiber depends on its polymerization and molecular weight, and this implies that not all soluble fibers are viscous fibers [5]. The most effective metabolic viscous fibers are psyllium, β-glucans, guar gum (galactomannan), glucomannan, and pectic polysaccharides [3,17]. However, even some non-viscous fibers, such as inulin-type fructans and manufactured low-viscosity, digestion-resistant maltodextrin, can lower glucose, insulin and LDL-cholesterol, while increasing HDL-cholesterol [5]. Nevertheless, fiber-rich foods, such as pulses, nuts, barley, oats, and some vegetables and fruits have shown cardio-metabolic protective effects. [5]. In general, soluble fibers are fermented by the gut microbiota, and the speed and degree of fermentation differs between fibers from different sources, between fibers used in different food combinations, when raw, between dry fibers and those prepared via cooking, and between fibers consumed with or without skin. Prebiotic fibers are greatly prevalent in chicory root, leeks, Jerusalem artichokes, asparagus, garlic, onions, wheat, oats, soybeans and bananas [12]. 

Carbohydrates with 3–20 sugar units are called oligosaccharides. Oligosaccharides such as lactose, sucrose and maltose are digested and absorbed in the small intestine, thus contributing to the energy source of the human body. Functional oligosaccharides, such as galacto-oligosaccharides (GOSs), fructo-oligosaccharides (FOSs), xylo-oligosaccharides, isomaltose and even human milk oligosaccharides reach the colon and act as prebiotics, particularly for bifidobacteria [18,19,20].

Fructans are non-digestible polymers of fructose and include oligofructose, inulin, inulin-type fructans, and FOSs. Fructans present beta (β) (2–1) fructosyl-fructose glycosidic bonds that are not digested by the enzymes of the human gut and may contain a terminal glucose. Fructans have varying degrees of polymerization (DP, number of sugar monomers in each chain), ranging between 2 and 60 DP. Short-chain fructooligosaccharides (sc-FOSs) have a DP of 3–6 units, oligofructose has a mean DP of 4, inulin has an average DP of 12, and long-chain FOSs (lcFOSs) and high-molecular-weight inulin have a mean DP of 25 [12]. Fructans occur naturally in plant-based foods such as chicory root, artichoke, wheat, onion, asparagus, agave, and banana. Inulin is commonly obtained via the hot water extraction of chicory root [12]. High-purity inulin is then produced via physical separation, whilst partial enzymatic hydrolysis via endoinulase is used to obtain oligofructose. Synthetic FOSs result from the enzymatic conversion of sucrose, commonly by exploiting the beta-fructosidase enzyme of the fungus *Aspergillus niger* [21]. FOS synthesis from glucose is also possible and produces short-chain inulin or FOSs (DP of 2–4) with a higher proportion of glucose [12]. The short-chain fructans (≤20 units) ferment faster than larger fructan molecules, such as some (long sugar chain) inulins [9]. 

GOSs have varying DP values and linkages to sugar monomers such as glucose and lactose. GOSs can be industrially synthesized through the enzymatic hydrolysis (β-galactosidase reaction) of the glycosidic bond present in lactose [12].

Resistant starches can be soluble or insoluble and show different intestinal fermentability, also depending on the composition of the gut microbiota that is, in turn, influenced by the intake of fibers [6]. Particularly, corn fiber is a soluble fiber with a mean DP of 10, has a low viscosity and is resistant to heat and variable pH. It is produced by the enzymatic hydrolysis of corn starch, resulting in glucose chains containing a mixture of α (1–2) (1–3) (1–4) (1–6) glycosidic linkages that are indigestible in the upper GI tract, thereby allowing for microbial fermentation in the lower gut [12]. 

Compared to refined grain, whole grains are considered to be a health-promoting nutrient for the presence of a bran, which is a fiber-filled outer layer also containing group B vitamins, minerals and antioxidants. Moreover, the whole grain has a germ (the core of the seed) that is rich in vitamin B and E, antioxidants and fats. By contrast, the milling process eliminates the bran and germ from grains and limits the fiber content to what is present in the more chewable and digestible endosperm (mainly arabinoxylan and β-glucan). Examples of whole grains include wheat bran, brown rice, barley, oats, corn and rye. 

Wheat bran contains the outer layers of the grain and is composed mainly of insoluble arabinoxylans and cellulose, but also of starch, proteins, beta-glucan, lignin, minerals, vitamins and lipids. The effect of consuming a wheat bran extract (5.0 g/day for 3 weeks) containing arabinoxylan-oligosaccharides was assessed in 29 healthy children (8–12 years). Fecal bifidobacteria tended to increase after wheat bran consumption (*p* = 0.069) and the bifidobacteria ratio to total fecal microbiota was significantly higher (*p* = 0.002) compared to the placebo group. A significant decrease in fecal isobutyric acid and isovaleric acid (*p* < 0.01), markers of protein fermentation, was detected. Wheat bran extract did not change the severity of flatulence, abdominal pain and the urge to vomit in this healthy population [22]. 

Beta-glucans (β-glucans) are non-starch polysaccharides of d-glucose monomers linked by β-glycosidic linkages. Beta-glucans are present in cereal grains, yeast, mushrooms, seaweeds, and some bacteria [12]. 

Arabinoxylans are non-starch polysaccharides found in many cereal grains. They consist of β(1,4) linked d-xylopyranosyl residues to which arabinofuranosyl moieties are attached. The partial enzymatic hydrolysis of arabinoxylans produces arabinoxylan oligosaccharides and xylooligosaccharides [12]. 

All carbohydrates that are not hydrolyzed by the human intestinal enzymes act as dietary fibers and when metabolized by the gut microbiota, produce SCFAs. However, the amount and rate of fermentation, pH drop, gas and SCFA production are a combination and effect of the quantity and type of fibers, individual gut microbiota, food matrix, cooking or other processes. Neither the chain length nor the degree of polymerization can predict the intestinal production of SCFAs [9]. SCFAs are represented by acetate, propionate and butyrate. An increasing body of evidence indicates the multiple health benefits of SCFAs, including as fuel for colonocytes, mucin production, the regulation of apoptosis and immunity, the modulation of satiety, the maintenance of intestinal barrier function, an increase in colonic mineral absorption (e.g., calcium, iron and magnesium), the stimulation of beneficial bacteria (e.g., bifidobacteria, lactobacilli), a reduction in inflammation and the development of immune tolerance [6,11,12]. While butyrate has a pivotal role in the gut, and has anti-inflammatory and immune effects, acetate and propionate regulate cholesterol biosynthesis and gluconeogenesis in the liver, adipocyte differentiation, metabolism and inflammation [6]. 

Infant formulas containing FOSs and/or GOSs may produce SCFAs and a gut microbiota rich in bifidobacteria, with similar effects to breastfeeding [23,24,25]. Moreover, disaccharides such as lactose, lactitol and lactulose, may exert prebiotic effects, whilst not all fibers produce SCFAs and affect gut microbiota [12]. 

Insoluble dietary fibers are represented by cellulose, hemicellulose, lignin, chitin and wheat bran. 

Insoluble fibers are mostly present in fruit (particularly in peel), in vegetable walls and roots, and in the whole grain. Chitins are long-chain polymers of N-acetylglucosamine and constitute the cell walls of fungi, and the exoskeleton of crustaceans and insects [10]. Insoluble fibers are largely not or poorly fermented, and therefore increase fecal bulk, stimulate bowel transit, facilitate the elimination of noxious substances and entrap possible pathogens. Whilst lignin and chitins do not modulate gut microbiota, certain cellulose, hemicellulose, xyloglucans and resistant starch may exert some prebiotic effects that can be enhanced by processing and biotechnology [6,10]. 

Xylitol is a non-digestible pentose sugar with an alcohol moiety that is found naturally in many fruits and vegetables and shows prebiotic effects. This prebiotic is manufactured via its extraction from lignocellulosic materials (polymers of cellulose, hemicellulose, and lignan), such as birchwood [12]. Xylooligosaccharides are non-digestible oligosaccharides with a mean DP of 2–6 and β(1–4) glycosidic bonds that also result from the direct enzymatic hydrolysis of xylan-rich lignocellulosic materials. Xylooligosaccharides present prebiotic effects [12].

Functional fibers include resistant starch, pectin, gums, polydextrose, inulin, and non-digestible dextrin or animal components (e.g., chitin and chitosan) that may be present or added to foods, or provided as supplements to confer health benefits [1,13].

Several fibers have been studied in children, particularly as a treatment for constipation [2,3,26,27,28] (Table 2). 

A number of studies have shown that the quality and health benefits of dietary fibers are determined by their different physical and chemical characteristics, combined with their fermentability and bulking properties. Since the last two effects have a relevant gut and extra-intestinal impact, a healthy diet should contain both soluble and insoluble fibers [6]. Many natural and added fibers are present in the diet and improve the nutritional value of foods [3]. Moreover, additional vegetable and fruit components (e.g., micronutrients, polyphenols, phytosterols, and phytoestrogens) may contribute to the health outcomes seen with the consumption of dietary fibers [5].

## 3. Fiber Requirement in Children

Many guidelines recommend a daily dietary fiber intake of 25–35 g for adults, whilst there is no consensus on the dietary fiber requirement of infants and children [3,6,10]. The American Academy of Pediatrics suggests two different formula by which to calculate the (minimum) daily fiber intake in pediatric subjects: one based on the age of the child (age in years + 5 g) and the other based on body weight (0.5 g fiber/kg up to 35 g/d). The American Health Foundation suggests to consume “age in years plus 5 g up to 10 g” of fiber per day for children over 2 years [33]. Other authorities, such as the Institute of Medicine and Food and Drug Administration, propose a dietary fiber requirement based on caloric intake (14 g or 12 g fiber/1000 kcal) [1]. Applying the different formulas to the same child would result in a widely different recommended intake, ranging from 7 to 19 g/day for young children and 20–38 g/d in school-age children and adolescents [1,6,10]. For infants and children under the age of 2 years, the optimal daily dietary fiber intake has not been established, but a variety of fruits, vegetables, and easily digested cereals have been introduced with complementary feeding. In adults, a ratio of 3:1 insoluble/soluble fibers is suggested [10], whilst no clear indication is available for the quality composition of fibers in children’s diets [6]. 

Since many studies have shown that the dietary fiber intake is low in most children consuming a Western diet, efforts to increase fiber consumption should be encouraged [1,6]. Barriers to dietary fiber intake include taste, visual appeal, cost and the limited availability of ready-to-eat foods for meals away from home [34,35].

## 4. Fibers in Functional Gastrointestinal Disorders in Children

Despite the lack of agreement regarding the recommended type and amount of fibers that have the best beneficial effect for children, evidence supports that dietary fibers contribute to the maintenance of healthy gastrointestinal functions and prevent childhood constipation [1,3]. However, there is a lack of large, randomized placebo-controlled trials evaluating the effectiveness of (different) fibers in children with functional gastrointestinal disorders (FGIDs). Moreover, the heterogeneity in the study design, type and dose of fibers, population enrolled, outcome measures and length of follow-up currently preclude the development of evidence-based recommendations for dietary fiber in the management of FGIDs in children [27,36].

## 5. Fibers in Constipation

Constipation is a common disorder affecting 3–29% of children worldwide, starting in many cases in the first year of life and persisting for years in 35–52% of subjects [27,37]. More than half of children with constipation have a fiber intake below the minimum recommendation, and a low-fiber diet has been associated with the development of constipation in children [4]. In a small prospective study, 21 (75%) children with a high bran and dietary fiber intake (>age + 10 g/day) recovered from constipation at the end of the intervention [38].

The ESPGHAN/NAPGHAN guideline recommends a normal amount of dietary fiber intake for children with functional constipation [27]. According to a synthesis of systematic reviews and meta-analyses, further supplementation with a normal (fiber containing) diet does not seem to improve constipation [39]. However, there are limited data and a lack of recommendations on the type or source of fiber that might be the most beneficial for these children [40]. 

In recent decades, different fibers have been tested to treat constipation in children in randomized control trials. They include cocoa husk, glucomannan, partially hydrolyzed guar gum, two herbs from traditional Persian Medicines (Cassia fistula and flixweed), FOSs, GOSs and different combinations of fibers (oligosaccharide, inulin, soybean fibers and starch; acacia and psyllium fibers plus fructose; fructooligosaccharides, inulin, Arabic gum, resistant starch, soy polysaccharide, and cellulose; polydextrose and fructooligosaccharide). 

In 97 children aged 3–10 years old with chronic idiopathic constipation, the intake of cocoa husk with milk (5.2 g 1–2/day) resulted in less hard stools compared to the control group (41.7 vs. 75.0%) [41]. 

Glucomannan (100 mg/kg/day with 50 mL of fluid) improved stool consistency in 31 children more frequently than maltodextrin (62% vs. 23%), and reduced infrequent bowel movements and abdominal pain, with a higher global improvement in constipation (42 vs. 13%) compared to the placebo group [42]. Likewise, glucomannan (100 mg 2 x/day), but not placebo, improved constipation in 10 children with severe brain damage and chronic constipation: stool frequency and consistency, the use of laxative or suppository, and episodes of painful defecation were significantly changed with fiber treatment (*p* < 0.01), despite no measurable effect on the total and segmental transit times [43]. In another cohort of 80 children, a different dose of glucomannan (1.26 g 2 x day with 125 mL of fluid) was not significantly more effective compared to maltodextrin in terms of the number of evacuations (≥3) per week with no soiling (relative risk 0.95, 95% CI 0.6 to 1.4), the stool consistency score at weeks 2 and 4, abdominal pain episodes at weeks 2 and 3, and other secondary outcomes or adverse events [44]. 

Partially hydrolyzed guar gum (3–5 g/day) added in fruit juice was tested vs. lactulose (1 mL/kg/day with juice) in 61 children. The bowel movement, stool consistency and number of children with abdominal pain improved significantly (*p* < 0.05) in both groups. Weekly defecation frequency increased more in the lactulose than in the guar gum group (from 4 ± 0.7 to 6 ± 1.06 and from 4 ± 0.7 to 5 ± 1.7, *p* < 0.05). However, the parents complained of bad taste and flatulence in the lactulose group [45].

Cassia fistula (Leguminosae) (0.1 g/kg/day) was compared to mineral oil (1 mL/kg/day) in 81 children. After three weeks of medication, more children in the fiber group improved (84% vs. 50%, *p* = 0.002), achieved a higher frequency of defecation per week (from 1.7 to 10.6 vs. from 2 to 6.1, *p* < 0.001), and experienced a reduced severity of pain during defecation and a changed consistency of stool (*p* < 0.05) compared to the control group. No significant differences were noted between the two groups in terms of fecal incontinence, retentive posturing and side effects [46]. 

Flixweed (Descurainia ydrol, rich in soluble and insoluble fiber) (2–3 g/day) was compared to polyethylene glycol (PEG 0.4 g/kg) in 120 children. At the end of the study, 64.3% patients in the flixweed and 54.7% in the PEG group were out of the Rome III criteria for constipation (*p* = 0.205). The median weekly stool frequency showed a similar improvement with both treatments. Flatulence was slightly less reported in the flixweed than in the PEG group (8.9% vs. 11.3%, *p* = 0.461), whereas taste was less accepted with flixweed [47]. 

In a double-blinded, placebo-controlled crossover trial, 20 children ingested GOSs (1.7 g/day) or maltodextrin for 30 days, followed by a 15-day washout period, and a 30-day period of the other treatment. GOSs were related to a more significant increase in bowel movement frequency, the relief of defecation straining, and a decrease in stool consistency (*p* < 0.0001) compared to the placebo [48].

A dietary fiber mixture of oligosaccharide, inulin, soybean fiber and starch (10 g/125 mL of yogurt, 1–3/day) was compared to lactulose (10 g/125 mL of yogurt, 1–3 x/day) in 135 children. At the end of the study, no difference was found between the groups in terms of the frequency of defecation and fecal incontinence, abdominal pain and flatulence scores, taste, adverse effects and step-up medications. The consistency of stools was softer in the lactulose group (*p* = 0.01) [49].

A mixture of acacia, psyllium, and fructose (16.8 g/day) was compared to PEG (0.5 g/kg/day) combined with electrolytes in 100 children. A similar improvement in constipation (77.8% vs. 83%) was detected in both groups after 8 weeks of treatment, with no clinically significant adverse effects. In this population, PEG was more accepted than fibers [50].

Fructooligosaccharides, inulin, Arabic gum, starch, soy polysaccharide, and cellulose (3.8–7.6 g 2×/day with 200 mL of chocolate milk) were compared to maltodextrin with chocolate milk in 54 children. Therapeutic failure was observed in approximately one third of the children in both groups. Children in the fiber group improved in terms of daily bowel movements and stool consistency significantly more than children in the maltodextrin group (0.53 vs. 0.23, *p* = 0.014) (60% vs. 16.7%, *p* = 0.003). The colonic transit time was similar between both arms [51]. 

In 2018, two different systematic reviews [28,52] concluded that some beneficial effects were observed with the use of fibers in children with constipation, but the different study designs, outcome assessments, definitions of therapeutic success and types of fibers used did not allow strong recommendations [28]. In 2022, a meta-analysis of 10 randomized control studies, including 690 children with functional constipation, evaluated the effect of seven different fiber mixtures [40]. A definition of treatment success was reported in 5 of 10 studies, among which, 1 (a mixture of acacia fiber, psyllium fiber, and fructose) was as effective as a laxative treatment [50], 1 (glucomannan) was more effective than the placebo [42], and 3 (glucomannan, fiber/prebiotic mixture [FOSs, inulin, gum Arabic, resistant starch, soy polysaccharide, and cellulose], FOSs in infant formula) were not more effective than the placebo [44,51,53]. The authors also highlighted that the type of fiber used in the studies perhaps did not reflect those most likely to benefit constipation (such as bulking fiber) [40]. 

Recently, a prospective study tested a mixture of polydextrose (4.17 g) and FOSs (0.45 g) in a daily dose of food supplement in 77 children. The number of children with less than three bowel movements per week dropped from 59.7% to 11.7%, hard stools (Bristol type 1 and 2) decreased from 68.8% to 7.8%, pain during defecation decreased from 79.2% to 10.4%, fear of defecation decreased from 68.8% to 3.9%, and the number of children with abdominal pain symptoms reduced from 84.2% to 2.6% at the end of the study. Noteworthy, there was no comparison with a control group [54]. 

A meta-analysis of fruit intervention for functional constipation has recently been conducted, including 11 studies in adult patients, showing that certain fruits may alleviate constipation, affecting stool consistency, the frequency of evacuation and gut microbiota. In particular, four trials showed that kiwifruits increased stool frequency (MD = 0.26, 95% CI (0.22, 0.30), *p* < 0.0001, *I*^2^ = 0%) more significantly than palm date or orange juices. Moreover, three high-quality studies found that kiwifruits have a better effect on stool symptoms and consistency (assessed by the Bristol stool scale) than Ficus carica paste [MD = 0.39, 95% CI (0.11, 0.66), *p* < 0.05, *I*^2^ = 27%]. Analyzing the prebiotic effects, five trials showed that fruits increased the amount of *Lactobacillus acidophilus* [MD = 0.82, 95% CI (0.25, 1.39), *p* < 0.05, *I*^2^ = 52%], and that pome fruits, citrus fruits, and berries increased bifidobacteria more than stone fruits [MD = 0.51, 95% CI (0.23, 0.79), *p* < 0.05, *I*^2^ = 84%] [55]. No similar data are currently available for children. 

In infants, softer stools and an increase in bifidobacteria have been reported in a number of clinical trials testing infant formulas containing a combination of β--palmitate, FOSs and GOSs [56,57,58,59]. An infant formula with GOSs at a low level (0.24 g/100 mL) improved stool frequency, decreased fecal pH, and increased bifidobacteria and lactobacilli in a similar manner to human milk and significantly more than a standard formula [60]. 

In 32 infants with constipation, the group fed a formula containing FOSs (DP from two to six monosaccharide molecules, dose 6, 9 or 12 g daily based on the infants’ weight groups) reported therapeutic success in 83.3% compared to in 55.6% of the control (maltodextrin) group (*p* = 0.073); this group also had a significantly higher frequency of softer stools (*p* = 0.035), fewer episodes of straining and/or difficulty passing stools (*p* = 0.041) and a shorter mouth-to-anus transit time (22.4 and 24.5 h, *p* = 0.035), with a higher Bifidobacterium count (*p* = 0.006) compared to the control group [53].

An infant formula with partial whey hydrolyzed proteins, starch, a high magnesium content, FOSs, GOSs and probiotics, reduced constipation in infants from 18.8% to 6.5% within three days [59].

However, different feeding components, such as the quantity and type of formula, hydrolyzed proteins, content of magnesium, characteristics of lipids, source, ratio and concentration of oligosaccharides, and possible additional probiotics, may contribute to an improvement in constipation in infants and make it difficult to extrapolate the effect of fibers in these trials [58,59,61,62]. A complete analysis of all the determinants of prebiotic and fiber efficacy in formula-fed constipated infants is beyond the scope of this review.

## 6. Fibers in Functional Abdominal Pain

The first trial on fibers for recurrent abdominal pain, defined with Apley’s criteria, dates back to 1985 [63]. Children (*n* = 26) supplemented for 2 weeks with two corn fiber cookies (5 g/cooky) per day demonstrated a halved frequency of attacks, compared to the ones given placebo (13 vs. 7, *p* = 0.04) [63]. In 2013, the administration of glucomannan (2.52 g/d, 1 sachet of 1.26 g in 125 mL of fluid 2 times a day) for 4 weeks in 84 children with functional abdominal pain according to the Rome III criteria was no more effective than the placebo [26]. In the same year, a partial hydrolyzed guar gum supplementation significantly reduced the intensity of abdominal pain compared to the placebo (40% vs. 13%, *p* = 0.025) [64]. In 2017, a Cochrane review on dietary intervention for recurrent or functional abdominal pain (according to Apley’s or Rome criteria) in childhood [Newlove-Delgado] included four RCTs [26,63,64,65] that assessed the effect of dietary fibers. These included corn fiber [63], partially hydrolyzed guar gum [64], glucomannan [26], and psyllium fiber (6–12 g/day) [65]. Children in the fiber groups showed a similar improvement in pain up to three months postintervention compared to children who received the placebo (OR 1.83, 95% CI 0.92 to 3.65; 2 studies; 136 children). There was also no reduction in pain intensity (SMD −1.24, 95% CI −3.41 to 0.94; 2 studies; 135 children). The evidence was judged to be of low quality due to the risk of bias, imprecision, and significant heterogeneity [36]. A 2022 systematic review and meta-analysis of dietary interventions for functional abdominal pain disorders in children did not identify any new pediatric study [66]. 

In 30 autistic children, a 6-week prebiotic intervention based on GOSs, combined with an exclusion (gluten and cow’s milk) diet, significantly decreased abdominal pain and bowel movement scores, the presence of bifidobacteria and veillonellaceae, and increased Faecalibacterium prausnitzii and bacteroides. In addition, after the intervention period, behavior improvement and significant changes in fecal and urine metabolites were also noted [67]. 

## 7. Fibers in Irritable Bowel Syndrome

Four studies have assessed the effect of fiber supplementation in children with IBS, classified according to the Rome criteria. A recent systematic review and meta-analysis [66] analyzed the results of three of them [64,65,68]. 

In 2013, partially hydrolyzed guar gum (5 g/day mixed in fruit juice) was supplemented in 60 children with chronic abdominal pain or IBS. Children in the fiber group reported a significant reduction in clinical symptoms compared to the control group (43% vs. 5%, *p* = 0.025), an improvement in the Birmingham IBS score (median 4 ± 1 vs. 0 ± 1, *p* = 0.025), and an improvement in stool consistency, evaluated with the Bristol Stool Scale (40% vs. 13.3%, *p* = 0.025) [64]. In another RCT including 71 children with IBS, treatment with inulin (900 mg, twice daily for 4 weeks) was less effective than treatment with probiotics and symbiotics [68]. In 2017, Shulman et al. found that the psyllium fiber (6 g for ages 7–11 years and 12 g for ages 12–17 years, for six weeks) lessened the mean number of pain episodes compared to the placebo (8.2 ± 1.2 vs. 4.1 ± 1.3, *p* = 0.03) in 84 children with IBS. Interestingly, pain intensity and gut microbiota did not differ between the groups [65]. 

Very recently, 4 weeks of psyllium supplementation (6–12 g/day, according to age) improved individuals’ score on the IBS severity scoring scale (IBS-SSS) more significantly compared to the placebo [75 (42.5–140) vs. 225 (185–270); *p* < 0.001], and showed a higher remission rate (IBS-SSS < 75) (43.9% vs. 9.7%, *p* < 0.0001) [69].

## 8. Fibers in Other Functional Gastrointestinal Disorders

To the best of our knowledge, no RCT has tested the effect of dietary fiber supplementation in children with abdominal migraine, functional dyspepsia, cyclic vomiting or rumination syndrome. 

In infantile regurgitation, corn or rice starch and locust bean gum have long been used in infant formulas as thickening agent in order to reduce the number of regurgitation episodes and the rate of regurgitating infants [62]. Locust bean gum presents the highest viscosity at the lowest concentration, but its clinical effect on regurgitation is similar to other thickening agents [70].

Different infant formulas containing short-chain GOSs and long-chain FOSs, partial or extensive hydrolyzed proteins, reduced lactose, modified fat content, and, in some trials, probiotics, have shown a significant reduction in crying, colic and regurgitation in selected infants [58,59,60,71,72]. However, the combination of these components may be responsible for the beneficial outcomes that cannot be attributed exclusively to the fiber content. A detailed analysis of all studies testing infant formulas with prebiotics in infants, possible confounders and determinants is beyond the scope of this review. A Cochrane review published in 2018 concluded that any dietary intervention must be evidence based to be recommended for infantile colic [73].

## 9. Fibers in Inflammatory Bowel Disease (IBD)

Dietary advice on the type and quantity of fibers in patients with IBD is currently uncertain, due to the limited data in adults [74] and lack of specific trials in children [75,76]. Many IBD patients limit their intake of fibers, particularly during active disease when fibers might be less tolerated and exacerbate symptoms. However, the possible beneficial effect of dietary fibers in these patients is being increasingly recognized. Specific dietary fibers can restore the mucus layer and barrier function, ameliorate dysbiosis, increase the production of gut SCFAs and microbial metabolites (particularly butyrate), and reduce inflammation [75]. In addition, the phenolic compounds of fruits, vegetables, whole grains, nuts and legumes may beneficially affect the gut microbiota and downregulate inflammatory pathways. High fruit consumption has been reported to be inversely related to the risk of IBD onset (both ulcerative colitis and Crohn’s disease), whilst vegetables are only inversely associated with ulcerative colitis. In contrast, in selected patients, certain fibers may induce pro-inflammatory cytokines, but this is largely dependent on baseline microbiota diversity [74]. A European pediatric consensus of experts concluded that, based on the current evidence, no dietary fiber restriction should be recommended to children with IBD without evidence of structuring phenotype or fiber intolerance [76]. 

A recent systematic review evaluated eight randomized control studies assessing five types of fibers in adult IBD patients [77]. Germinated barley foodstuff significantly decreased pro-inflammatory cytokines (TNF-alfa and IL-6) in patients with ulcerative colitis in remission, and reduced clinical and endoscopic disease activity scores in another group of patients with mild to moderate active colitis. Ispaghula (psyllium) husk lowered symptoms in adult patients with ulcerative colitis in remission, although the reduction rate was not significantly different compared to the placebo. Wheat bran plus psyllium beneficially affected intestinal microbiota, but not fecal mass, in a group of patients with ulcerative colitis in remission. In another trial, wheat bran plus resistant starch did not show a difference in terms of disease activity, symptoms, fecal mass and metabolites. Plantago ovata seeds increased butyrate in patients with ulcerative colitis in remission without a significant difference in terms of remaining in remission compared to the control group. Inulin was supplemented in patients with mild to moderate active ulcerative colitis: dyspeptic symptoms and fecal calprotectin reduced significantly after one week of intervention, but no difference was reported in the quality of life and disease activity. The only study enrolling patients with Crohn’s disease found that FOSs did not significantly modify disease activity and fecal calprotectin, and instead lowered the quality of life scores. Only one study included in this review was considered as having a low risk of bias [77]. 

The fiber intake of pediatric IBD patients is suboptimal and often lower than in children without IBD [75]. To date, there is very limited evidence regarding the effectiveness of supplemented dietary fibers in (adult) ulcerative colitis in remission, and conflicting results have been reported in the active phase; in addition, no clear suggestions have been made for individuals with Crohn’s disease and pediatric patients [76,77]. Moreover, data in adults are limited by the small sample size, short duration, high dropout rate, different type, dose and duration of fiber intervention, disease activity and outcome scores. 

## 10. Adverse Effects of Fibers 

In all clinical trials assessing the effects of fibers in children, no serious adverse events have been reported and the products have been well tolerated [20,22,27,28,40,51,62,63,64,65,66,67,68,69,70,71,72,73]. In four studies, mild side effects such as diarrhea, abdominal distention, flatulence, and vomiting were observed in the experimental group [44,49,50,53]. Since fibers are resistant to digestion and not absorbed in the human small intestine, partial or complete fermentation occurs in the large intestine, creating flatulence, diarrhea, abdominal distension and discomfort, depending on the quantity and type of fibers, other food components, gut microbiota, and individual’s response and sensitivity [1,10,12,78,79,80,81,82]. Nonetheless, cooking, the food matrix, the glucose/fructose ratio, and the simultaneous intake of other fermentable carbohydrates or f polyols (FODMAP content) may interfere or contribute to intolerance or adverse effects [78,79,80,81]. Moreover, age, the host microbiota composition, inflammation, recent infections and stress conditions may also have an impact on the intestinal response [80,83]. Commercial anti-regurgitation infant formulas must have a controlled composition, with thickening components that are less than 2 g/100 mL for starch and 1 g/100 mL for carob bean gum, in order to avoid excessive fermentation, diarrhea and an impaired mineral bioavailability [70]. Excessive fiber intake during childhood should be avoided in order to limit intestinal fermentation, a possible inadequate energy intake due to increased satiety, and the reduced bioavailability of minerals. Many sources of fiber are high in phytates and oxalates, which decrease iron bioavailability [84]. In contrast, SCFAs and a decreased intestinal pH may favor the absorption of minerals such as iron, calcium and magnesium [6,11,12].

## 11. Conclusions

The physical and chemical characteristics of fibers, combined with their fermentability and bulking properties, determine the health effects of different dietary fibers. Children should ingest a variety of fibers from fruits, vegetables, legumes, seeds, nuts and cereal grains to ensure and achieve the benefits and synergies of different dietary fibers. A few pediatric studies have shown the beneficial effect of certain fiber or various combinations of fibers on functional constipation, abdominal pain and irritable bowel syndrome. The small sample size and heterogeneity in the study design, in the population recruited, in the type and dose of fibers, and in the outcome measures, currently preclude the recommendation of a specific dietary fiber for children with gastrointestinal disorders. 

## Figures and Tables

**Table 1:** Content and types of fiber in common food items.

A. Content of fibers in common fruits, vegetables and nuts							
**FOOD** 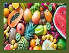	**Total** **Fibers g/100 g**	**Soluble Fiber g/100 g**	**Insoluble Fiber** **g/100 g**	**FOOD** 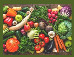	**Total** **Fibers g/100 g**	**Soluble Fiber g/100 g**	**Insoluble Fiber** **g/100 g**
**FRUITS**				VEGETABLES			
**Apple (with skin)**	2.6	0.73	1.84	Artichoke (frozen)	5.0	3.04	1.93
**Apple (without skin)**	2.0	1.55	0.44	Asparagus	3.5	2.6	0.9
**Apricot**	1.5	0.71	0.83	Aubergine	2.5	0.6	1.9
**Avocado**	6.7	4.7	2.0	Beetroot	7.8	2.4	5.4
**Banana**	1.8	0.62	1.19	Broccoli	2.4	0	2.4
**Blueberry (bilberry)**	3.3	0.7	2.6	Brussels sprouts	4.4	2.8	1.6
**Cherry**	1.3	0.49	0.8	Cabbage (green)	2.6	0.32	2.26
**Coconut**	9	0.5	8.5	Cabbage (red)	2.5	1.4	1.1
**Grape (black)**	1.6	0.25	1.33	Cabbage (savoy)	2.9	0.35	2.53
**Grape (white)**	1.4	0.16	1.2	Carrot	3.1	0.41	2.7
**Grapefruit**	1.6	0.54	1.06	Cauliflower	1.9	1.4	0.5
**Kiwi**	2.2	0.78	1.43	Celeriac	2.9	0.9	2.0
**Lemon**	2.8	1.6	1.2	Celery	1.6	0.18	1.41
**Mandarin**	1.9	0.9	1.0	Cucumber	0.8	0.21	0.54
**Mango**	2.0	1.0	1.0	Eggplant	6.6	1.3	5.3
**Melon (cantaloupe)**	0.8	0.3	0.5	Fennel	2.2	0.25	1.97
**Melon (honeydew)**	0.8	0.4	0.4	Green beans	2.9	0.71	2.14
**Olive (in oil)**	4.4	1.8	2.6	Italian chicory	3.0	0.59	2.37
**Orange (generic)**	1.6	0.6	1.0	Lettuce	1.5	0.13	1.33
**Peach (with skin)**	1.9	0.78	1.19	Mushrooms (boletus)	1.9	0.1	1.8
**Peach (without skin)**	1.6	0.87	0.71	Mushrooms (field)	2.3	0.11	2.14
**Pear (with skin)**	4.3	4.0	0.3	Mushrooms (pleurotes)	2.4	0.25	2.15
**Pear (without skin)**	3.8	1.29	2.56	Onion	1.0	0.16	0.88
**Pineapple**	1.0	0.15	0.83	Pepper	1.9	0.43	1.47
**Plum (red)**	1.6	0.67	0.91	Potato (boiled—with skin)	1.6	0.71	0.85
**Plum (yellow)**	1.4	0.57	0.83	Potato (raw—with skin)	1.8	0.8	0.96
**Raspberry**	3.7	0.4	3.3	Potato (raw)	1.3	0.3	1.0
**Strawberry**	1.6	0.45	1.13	Pumpkin	2.0	0.6	1.4
**Tangerine**	1.7	0.67	1.03	Radish	1.3	0.07	1.23
**Watermelon**	0.2	0.02	0.2	Spinach (fresh—raw)	2.6	0.5	2.1
NUTS				Spinach (frozen)	1.3	0.4	0.9
**Almond**	11.2	1.1	10.1	**Sweetcorn (boiled)**	3.5	0.3	3.2
**Brazil Nut**	6.5	5.1	1.4	**Sweetcorn (kernels)**	3.7	0.3	3.4
**Cashew Nut**	3.5	1.6	1.9	Tomato (canned)	1.9	1.7	0.2
**Hazelnut (dried)**	17.5	11.8	5.7	Tomato (fresh)	1.0	0.24	0.77
**Peanut (roasted)**	10.9	1	9.9	Tomato (puree)	1.0	0.6	0.4
**Pecan nuts**	9.4	0.3	9.1	Turnip	2.6	0.29	2.32
**Pine nuts**	4.6	0.8	3.8				
**Walnuts**	6.7	1.7	5.0				
**Chestnut**	4.7	0.4	4.3				
B. Content of fibers in grains and legumes							
**FOOD** 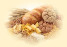	**Total** **Fibers g/100 g**	**Soluble Fiber g/100 g**	**Insoluble Fiber** **g/100 g**	**FOOD** 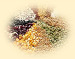	**Total** **Fibers g/100 g**	**Soluble Fiber g/100 g**	**Insoluble Fiber** **g/100 g**
**GRAINS**				**GRAINS**			
**Barley (pearled)**	9.2	4.41	4.83	**Rice (brown)**	4.0	0.1	3.9
**Biscuits (for children)**	1.3	0.3	1.0	**Rice (generic)**	1.3	0.3	1.0
**Biscuits (dried)**	2.6	1.32	1.32	**Rice (white—long grain—parboiled)**	1.7	0.7	1.0
**Biscuits (whole wheat)**	6.0	0.94	5.07	**Rice cake**	7.4	1.1	6.3
**Bread (flour type 00)**	3.1	1.46	1.63	**Rusks**	3.5	0.8	2.7
**Bread (whole wheat)**	6.5	1.15	5.36	**Rusks (whole wheat)**	4.3	0.8	3.5
**Corn flakes**	2.7	1.0	1.7	**Semolina**	3.5	0.9	2.6
**Corn flakes (sugar coated)**	1.8	0.7	1.1	**Spelt**	6.5	0.96	5.5
**Cornflour**	3.1	0.35	2.76	**Maize**	12.8	1.6	11.2
**Cornstarch**	0	0	0	Quality Protein Maize (QPM)	14.9	1.1	13.8
**Couscous**	5.0	2.4	2.6	**LEGUMES**			
**Crackers (salted)**	2.7	0.7	2.0	Beans (cannellini—cooked)	7.8	1.05	6.78
**Crackers (wholewheat)**	10.0	1.2	8.8	Beans (cannellini—dry weight)	17.6	2.3	15.25
**Egg pasta (fresh) (f)**	3.6	1.7	1.9	Beans (generic—cooked)	7.8	1.05	6.78
**Flour (wheat type 0)**	2.9	1.07	1.86	Beans (generic—dry weight)	17.5	2.34	15.14
**Flour (wheat type 00)**	2.2	0.84	1.41	Beans (pinto—cooked)	6.9	0.81	6.28
**Flour (wheat whole wheat)**	8.4	1.92	6.51	Beans (pinto—dry weight)	17.3	1.54	15.71
**Millet (flour)**	3.2	0.6	2.6	Chickpeas (dry weight)	13.6	1.13	12.45
**Muesli**	8.7	3.3	5.4	Chickpeas (cooked)	5.8	0.47	5.29
**Oat flakes**	8.3	3.3	4.99	Fava beans (shelled—cooked)	3.0	0.24	2.89
**Oat flour**	9.0	4.4	4.6	Fava beans (shelled—dry weight)	7.0	0.36	6.64
**Pasta (durum wheat flour)**	1.7	0.72	0.98	Lentils (cooked)	8.3	0.53	7.74
**Pasta (whole wheat, durum wheat)**	7.1	1.53	5.57	Lentils (dry weight)	13.8	0.92	12.91
**Quinoa (cooked)**	2.8	2.8	0	Peas (cooked)	6.4	0.64	5.73
**Rice (white)**	2.3	0.2	2.1	Peas (frozen)	3.5	0.3	3.2
**Rice (whole grain)**	7.4	1.1	6.3	Soybeans (dry weight)	15.7	6.4	9.3

**Table 2:** Type and characteristics of the fibers most commonly assessed in pediatric clinical trials.

FIBER	TYPE	SOURCE	COMPONENTS	PREBIOTIC EFFECT	Reference
**Glucomannan**	Soluble	Japanese konjac plant	Polysaccharide of 1,4-d-glucose and d-mannose	YES	[26]
**Acacia/Arabic gum**	Soluble	Acacia trees (Leguminosae)	Anionic polysaccharide: l-arabinose, l-rhamnose, and d-glucuronic acid and 1,3-linked β-d-galactopyranosyl units	YES	[2,3]
**Arabinoxylan**	Soluble	Whole grains (endosperm and bran)	Hemicellulose, polymers of two pentose sugars: arabinose and 1,4-linked xylose units	YES	[3,29]
**Beta-glucan**	Soluble	endosperm of barley and oats	Glucose polysaccharide with Beta (1–4) (1–3) linkages ± branch points	YES	[3,12,17]
**Bran**	Soluble (oat bran); insoluble (wheat, rice, corn, bran)	outermost layer cereal grains	Non-starch polysaccharides, cellulose, hemicellulose, lignin	YES, as source of arabinoxylan-oligosaccharides (i.e., wheat bran)	[2,3]
**Fructo-oligosaccharides (FOSs)**	Soluble	many plants (i.e., garlic, chicory, onion, artichoke, and banana	Linear chains of fructose units linked by beta bonds. The number of fructose units ranges from 2 to 60 and often ends in a terminal glucose unit.	YES (>>Bifidobacteria)	[3,23,30]
**Galactooligosaccharides (GOS)**	Soluble	Dairy products, beans, certain root vegetables	They are produced commercially from lactose by β-galactosidase	YES	[2,20,23,24,31]
**Inulin**	Soluble	Chicory roots, artichokes, bananas, onion, garlic, and wheat	Fructose polymers (ranging from 2 to >60) that are linked by beta bonds and that terminate with a glucose unit	YES	[29,32]
**Partially hydrolyzed guar gum**	Soluble	Extracted from guar beans from the Cyamopsis tetragonolobus plant	High-molecular-weight polysaccharide: galactomannans of a linear chain of (1-4)-linked β-d-mannopyranosyl units with (1-6)-linked α-d-galactopyranosyl residues as side chains. The mannose: galactose ratio is approximately 2:1.	YES	[2,3,10]
**Resistant starch**	Soluble	Grains, starch or chemically modified starch	High-molecular carbohydrate: linear (amylose) and branched (amylopectin) chains of glucose residues. RS1 is a physically protected form of starch found in whole or partly milled grains; RS2 is present as raw granules; RS3 is retrograded starch, composed of crystallized starches produced via unique cooking and cooling processes; and RS4 is a chemically modified starch.	YES	[2,3,29]
**Dextrin**	Soluble	Any starch source (corn, wheat, potatoes). There are as follows: white dextrin, yellow or canary dextrin, or British gums	Saccharide polymer linked primarily by alpha-(1 --> 4) d-glucose units and prepared by partial hydrolysis of starch	YES	[2]
**Psyllium**	Soluble	Seeds of the plant genus Plantago	Highly branched arabinoxylan	YES	[2]

## References

[1] Kranz S., Brauchla M., Slavin J.L., Miller K.B. (2012). What do we know about dietary fiber intake in children and health? The effects of fiber intake on constipation, obesity, and diabetes in children. Adv. Nutr..

[2] Slavin J. (2013). Fiber and prebiotics: Mechanisms and health benefits. Nutrients.

[3] Korczak R., Kamil A., Fleige L., Donovan S.M., Slavin J.L. (2017). Dietary fiber and digestive health in children. Nutr. Rev..

[4] Rajindrajith S., Devanarayana N.M., Benninga M.A. (2022). Childhood constipation: Current status, challenges, and future perspectives. World J. Clin. Pediatr..

[5] Augustin L.S.A., Aas A.-M., Astrup A., Atkinson F.S., Baer-Sinnott S., Barclay A.W., Brand-Miller J.C., Brighenti F., Bullo M., Buyken A.E. (2020). Dietary fibre consensus from the International carbohydrate quality Consortium (ICQC). Nutrients.

[6] Hojsak I., Benninga M.A., Hauser B., Kansu A., Kelly V.B., Stephen A.M., Morais Lopez A., Slavin J., Tuohy K. (2022). Benefits of dietary fibre for children in health and disease. Arch. Dis. Child..

[7] Burkitt D.P. (1973). Some diseases characteristic of modern Western civilization. Br. Med. J..

[8] Hipsley E.H. (1953). Dietary “fibre” and pregnancy toxaemia. Br. Med. J..

[9] Joint FAO/WHO Food Standards Programme, Secretariat of the CODEX Alimentarius Commission (2010). CODEX Alimentarius (CODEX) Guidelines on Nutrition Labeling CAC/GL 2–1985 as Last Amended 2010.

[10] Stephen A.M., Champ M.M., Cloran S.J., Fleith M., van Lieshout L., Mejborn H., Burley V.J. (2017). Dietary fibre in Europe: Current state of knowledge on definitions, sources, recommendations, intakes and relationships to health. Nutr. Res. Rev..

[11] Scholz-Ahrens K.E., Ade P., Marten B., Weber P., Timm W., Asil Y., Glüer C.-C., Schrezenmeir J. (2007). Prebiotics, probiotics, and synbiotics affect mineral absorption, bone mineral content, and bone structure. J. Nutr..

[12] Whisner C.M., Castillo L.F. (2018). Prebiotics, Bone and Mineral Metabolism. Calcif. Tissue Int..

[13] Trumbo P., Schlicker S., Yates A.A., Poos M., Food and Nutrition Board of the Institute of Medicine, The National Academies (2002). Dietary reference intakes for energy, carbohydrate, fiber, fat, fatty acids, cholesterol, protein and amino acids. J. Am. Diet. Assoc..

[14] Gibson G.R., Hutkins R., Sanders M.E., Prescott S.L., Reimer R.A., Salminen S.J., Scott K., Stanton C., Swanson K.S., Cani P.D. (2017). Expert consensus document: The International Scientific Association for Probiotics and Prebiotics (ISAPP) consensus statement on the definition and scope of prebiotics. Nat. Rev. Gastroenterol. Hepatol..

[15] Lee S.C., Prosky L., DeVries J.W. (1992). Determination of total, soluble and insoluble dietary fiber in foodsenzymatic-gravimetric method, MES-tris buffer: Collaborative study. JAOAC Int..

[16] Dhingra D.R., Michael M., Rajput H., Patil R.T. (2012). Dietary fibre in foods: A review. J. Food Sci. Technol..

[17] Tosh S.M. (2013). Review of human studies investigating the post-prandial blood-glucose lowering ability of oat and barley food products. Eur. J. Clin. Nutr..

[18] Coppa G.V., Bruni S., Morelli L., Soldi S., Gabrielli O. (2004). The first prebiotics in humans: Human milk oligosaccharides. J. Clin. Gastroenterol..

[19] Bode L. (2009). Human milk oligosaccharides: Prebiotics and beyond. Nutr. Rev..

[20] Mei Z., Yuan J., Li D. (2022). Biological activity of galacto-oligosaccharides: A review. Front. Microbiol..

[21] Kelly G. (2008). Inulin-type p.rebiotics—A review: Part 1. Altern. Med. Rev. J. Clin. Ther..

[22] François I.E., Lescroart O., Veraverbeke W.S., Marzorati M., Possemiers S., Hamer H., Windey K., Welling G.W., Delcour J.A., Courtin C.M. (2014). Effects of wheat bran extract containing arabinoxylan oligosaccharides on gastrointestinal parameters in healthy preadolescent children. J. Pediatr. Gastroenterol. Nutr..

[23] Moro G., Minoli I., Mosca M., Fanaro S., Jelinek J., Stahl B. (2002). Dosage-related bifidogenic effects of galacto- and fructooligosaccharides in formula-fed term infants. J. Pediatr. Gastroenterol. Nutr..

[24] Boehm G., Lidestri M., Casetta P., Jelinek J., Negretti F., Stahl B., Marini A. (2002). Supplementation of a bovine milk formula with an oligosaccharide mixture increases counts of faecal bifidobacteria in preterm infants. Arch. Dis. Childhood. Fetal Neonatal Ed..

[25] Knol J., Scholtens P., Kafka C., Steenbakkers J., Gro S., Helm K., Klarczyk M., Schöpfer H., Böckler H.-M., Wells J. (2005). Colon microflora in infants fed formula with galacto- and fructo-oligosaccharides: More like breast-fed infants. J. Pediatr. Gastroenterol. Nutr..

[26] Horvath A., Dziechciarz P., Szajewska H. (2013). Glucomannan for abdominal pain-related functional gastrointestinal disorders in children: A randomized trial. World J. Gastroenterol..

[27] Tabbers M.M., DiLorenzo C., Berger Y.M., Faure C., Langendam W.M., Nurko S., Staiano A., Vandenplas Y., Benninga A.M. (2014). Evaluation and treatment of functional constipation in infants and children: Evidence-based recommendations from ESPGHAN and NASPGHAN. J. Pediatr. Gastroenterol. Nutr..

[28] Axelrod C.H., Saps M. (2018). The Role of Fiber in the Treatment of Functional Gastrointestinal Disorders in Children. Nutrients.

[29] Lattimer J.M., Haub M.D. (2010). Effects of dietary fiber and its components on metabolic health. Nutrients.

[30] Sabater-Molina M., Larque E., Torrella F., Zamora S. (2009). Dietary fructooligosaccharides and potential benefits on health. J. Physiol. Biochem..

[31] Niittynen L., Kajander K., Korpela R. (2007). Galacto-oligosaccharides and bowel function. Scand. J. Food Nutr..

[32] Gibson G.R., Beatty E.R., Wang X., Cummings J.H. (1995). Selective stimulation of bifidobacteria in the human colon by oligofructose and inulin. Gastroenterology.

[33] Williams C.L., Bollella M., Wynder E.L. (1995). A new recommendation for dietary fiber in childhood. Pediatrics..

[34] Kamar M., Evans C., Hugh-Jones S. (2016). Factors influencing adolescent whole grain intake: A theory-based qualitative study. Appetite.

[35] Larson N.I., Neumark-Sztainer D., Story M., Burgess-Champoux T. (2010). Whole-grain intake correlates among adolescents and young adults: Findings from Project EAT. J. Am. Diet. Assoc..

[36] Newlove-Delgado T.V., Martin A.E., Abbott R.A., Bethel A., Thompson-Coon J., Whear R., Logan S. (2017). Dietary interventions for recurrent abdominal pain in childhood. Cochrane Database Syst. Rev..

[37] Connor F., Salvatore S., D’Auria E., Baldassarre M.E., Acunzo M., Di Bella G., Farella I., Sestito S., Pensabene L. (2022). Cows’ Milk Allergy-Associated Constipation: When to Look for It? A Narrative Review. Nutrients.

[38] Maffei Leoni H.V., Vicentini A.P. (2011). Prospective evaluation of dietary treatment in childhood constipation: High dietary fiber and wheat bran intake are associated with constipation amelioration. J. Pediatr. Gastroenterol. Nutr..

[39] Southwell B.R. (2020). Treatment of childhood constipation: A synthesis of systematic reviews and meta-analyses. Expert Rev. Gastroenterol. Hepatol..

[40] Wegh C.A.M., Baaleman D.F., Tabbers M.M., Smidt H., Benninga M.A. (2022). Nonpharmacologic Treatment for Children with Functional Constipation: A Systematic Review and Meta-analysis. J. Pediatr..

[41] Castillejo G., Bullo M., Anguera A., Escribano J., Salas-Salvado J. (2006). A controlled, randomized, double-blind trial to evaluate the effect of a supplement of cocoa husk that is rich in dietary fiber on colonic transit in constipated pediatric patients. Pediatrics.

[42] Loening-Baucke V., Miele E., Staiano A. (2004). Fiber (glucomannan) is beneficial in the treatment of childhood constipation. Pediatrics.

[43] Staiano A., Simeone D., Del Giudice E., Miele E., Tozzi A., Toraldo C. (2000). Effect of the dietary fiber glucomannan on chronic constipation in neurologically impaired children. J. Pediatr..

[44] Chmielewska A., Horvath A., Dziechciarz P., Szajewska H. (2011). Glucomannan is not effective for the treatment of functional constipation in children: A double-blind, placebo-controlled, randomized trial. Clin. Nutr..

[45] Üstündağ G., Kuloğlu Z., Kirbaş N., Kansu A. (2010). Can partially hydrolyzed guar gum be an alternative to lactulose in treatment of childhood constipation?. Turk. J. Gastroenterol. Off. J. Turk. Soc. Gastroenterol..

[46] Mozaffarpur S.A., Naseri M., Esmaeilidooki M.R., Kamalinejad M., Bijani A. (2012). The effect of cassia fistula emulsion on pediatric functional constipation in comparison with mineral oil: A randomized, clinical trial. Daru J. Fac. Pharm. Tehran Univ. Med. Sci..

[47] Nimrouzi M., Zarshenas M.M. (2015). Functional constipation in children: Non-pharmacological approach. J. Integr. Med..

[48] Beleli C.A.V., Antonio M.A., dos Santos R., Pastore G.M., Lomazi E.A. (2015). Effect of 4’galactooligosaccharide on constipation symptoms. J. Pediatr..

[49] Kokke F.T., Scholtens P.A., Alles M.S., Decates T.S., Fiselier T.J., Tolboom J.J., Kimpen J.L., Benninga M.A. (2008). A dietary fiber mixture versus lactulose in the treatment of childhood constipation: A double-blind randomized controlled trial. J. Pediatr. Gastroenterol. Nutr..

[50] Quitadamo P., Coccorullo P., Giannetti E., Romano C., Chiaro A., Campanozzi A., Poli E., Cucchiara S., Di Nardo G., Staiano A. (2012). A randomized, prospective, comparison study of a mixture of acacia fiber, psyllium fiber, and fructose vs. polyethylene glycol 3350 with electrolytes for the treatment of chronic functional constipation in childhood. J. Pediatr..

[51] Weber T.K., Toporovski M.S., Tahan S., Neufeld C.B., de Morais M.B. (2014). Dietary fiber mixture in pediatric patients with controlled chronic constipation. J. Pediatr. Gastroenterol. Nutr..

[52] Piccoli de Mello P., Eifer D.A., Daniel de Mello E. (2018). Use of fibers in childhood constipation treatment: Systematic review with meta-analysis. J. Pediatr..

[53] Souza D.D.S., Tahan S., Weber T.K., Araujo-Filho H.B., De Morais M.B. (2018). Randomized, Double-Blind, Placebo-Controlled Parallel Clinical Trial Assessing the Effect of Fructooligosaccharides in Infants with Constipation. Nutrients.

[54] Toporovski M.S., de Morais M.B., Abuhab A., Crippa J.M.A. (2021). Effect of Polydextrose/Fructooligosaccharide Mixture on Constipation Symptoms in Children Aged 4 to 8 Years. Nutrients.

[55] Huo J., Wu L., Lv J., Cao H., Gao Q. (2022). Effect of fruit intake on functional constipation: A systematic review and meta-analysis of randomized and crossover studies. Front. Nutr..

[56] Bongers M.E., de Lorijn F., Reitsma J.B., Groeneweg M., Taminiau J.A., Benninga M.A. (2007). The clinical effect of a new infant formula in term infants with constipation: A double-blind, randomized cross-over trial. Nutr. J..

[57] Vivatvakin B., Mahayosnond A., Theamboonlers A., Steenhout P.G., Conus N.J. (2010). Effect of a whey-predominant starter formula containing LCPUFAs and oligosaccharides (FOS/GOS) on gastrointestinal comfort in infants. Asia Pac. J. Clin. Nutr..

[58] Vandenplas Y., Benninga M., Broekaert I., Falconer J., Gottrand F., Guarino A., Lifschitz C., Lionetti P., Orel R., Papadopoulou A. (2016). Functional gastro-intestinal disorder algorithms focus on early recognition, parental reassurance and nutritional strategies. Acta Paediatr..

[59] Vandenplas Y., Gerlier L., Caekelbergh K., Possner M., Nan-Study-Group (2021). An Observational Real-Life Study with a New Infant Formula in Infants with Functional Gastro-Intestinal Disorders. Nutrients.

[60] Ben X.M., Li J., Feng Z.T., Shi S.Y., Lu Y.D., Chen R., Zhou X.Y. (2008). Low level of galacto-oligosaccharide in infant formula stimulates growth of intestinal Bifidobacteria and Lactobacilli. World J. Gastroenterol..

[61] Salvatore S. (2007). Nutritional options for infant constipation. Nutrition.

[62] Salvatore S., Abkari A., Cai W., Catto-Smith A., Cruchet S., Gottrand F., Hegar B., Lifschitz C., Ludwig T., Shah N. (2018). Review shows that parental reassurance and nutritional advice help to optimise the management of functional gastrointestinal disorders in infants. Acta Paediatr..

[63] Feldman W., McGrath P., Hodgson C., Ritter H., Shipman R.T. (1985). The use of dietary fiber in the management of simple, childhood, idiopathic, recurrent, abdominal pain. Results in a prospective, double-blind, randomized, controlled trial. Am. J. Dis. Child..

[64] Romano C., Comito D., Famiani A., Calamara S., Loddo I. (2013). Partially hydrolyzed guar gum in pediatric functional abdominal pain. World J. Gastroenterol..

[65] Shulman R.J., Hollister E.B., Cain K., Czyzewski D.I., Self M.M., Weidler E.M., Devaraj S., Luna R.A., Versalovic J., Heitkemper M. (2017). Psyllium Fiber Reduces Abdominal Pain in Children With Irritable Bowel Syndrome in a Randomized, Double-Blind Trial. Clin. Gastroenterol. Hepatol..

[66] de Bruijn C.M., Rexwinkel R., Gordon M., Sinopoulou V., Benninga M.A., Tabbers M.M. (2022). Dietary interventions for functional abdominal pain disorders in children: A systematic review and meta-analysis. Expert Rev. Gastroenterol. Hepatol..

[67] Grimaldi R., Gibson G.R., Vulevic J., Giallourou N., Castro-Mejia J.L., Hansen L.H., Gibson E.L., Nielsen D.S., Costabile A. (2018). A prebiotic intervention study in children with autism spectrum disorders (ASDs). Microbiome.

[68] Basturk A., Artan R., Yilmaz A. (2016). Efficacy of synbiotic, probiotic, and prebiotic treatments for irritable bowel syndrome in children: A randomized controlled trial. Turk. J. Gastroenterol..

[69] Menon J., Thapa B.R., Kumari R., Puttaiah Kadyada S., Rana S., Lal S.B. (2023). Efficacy of Oral Psyllium in Pediatric Irritable Bowel Syndrome: A Double-Blind Randomized Control Trial. J. Pediatr. Gastroenterol. Nutr..

[70] Salvatore S., Savino F., Singendonk M., Tabbers M., Benninga M.A., Staiano A., Vandenplas Y. (2018). Thickened infant formula: What to know. Nutrition.

[71] Savino F., Palumeri E., Castagno E., Cresi F., Dalmasso P., Cavallo F. (2006). Reduction of crying episodes owing to infantile colic: A randomized controlled study on the efficacy of a new infant formula. Eur. J. Clin. Nutr..

[72] Vandenplas Y., Ludwig T., Bouritius H. (2014). The combination of scGOS/lcFOS with fermented infant formula reduces the incidence of colic in 4 week old infants. Arch. Dis. Child..

[73] Gordon M., Biagioli E., Sorrenti M., Lingua C., Moja L., Banks S.S., Ceratto S., Savino F. (2018). Dietary modifications for infantile colic. Cochrane Database Syst. Rev..

[74] Haskey N., Gold S.L., Faith J.J., Raman M. (2023). To Fiber or Not to Fiber: The Swinging Pendulum of Fiber Supplementation in Patients with Inflammatory Bowel Disease. Nutrients.

[75] Healey G.R., Celiberto L.S., Lee S.M., Jacobson K. (2020). Fiber and Prebiotic Interventions in Pediatric Inflammatory Bowel Disease: What Role Does the Gut Microbiome Play?. Nutrients.

[76] Miele E., Shamir R., Aloi M., Assa A., Braegger C., Bronsky J., de Ridder L., Escher J.C., Hojsak I., Kolaček S. (2018). Nutrition in Pediatric Inflammatory Bowel Disease: A Position Paper on Behalf of the Porto Inflammatory Bowel Disease Group of the European Society of Pediatric Gastroenterology, Hepatology and Nutrition. J. Pediatr. Gastroenterol. Nutr..

[77] Peters V., Dijkstra G., Campmans-Kuijpers M.J.E. (2022). Are all dietary fibers equal for patients with inflammatory bowel disease? A systematic review of randomized controlled trials. Nutr. Rev..

[78] Giorgio V., Margiotta G., Stella G., Di Cicco F., Leoni C., Proli F., Zampino G., Gasbarrini A., Onesimo R. (2022). Intestinal Permeability in Children with Functional Gastrointestinal Disorders: The Effects of Diet. Nutrients.

[79] Turco R., Salvatore S., Miele E., Romano C., Marseglia G.L., Staiano A. (2018). Does a low FODMAPsdiet reduce symptoms of functional abdominal pain disorders? A systematic review in adult and paediatric population, on behalf of Italian Society of Pediatrics. Ital. J. Pediatr..

[80] Pensabene L., Salvatore S., Turco R., Tarsitano F., Concolino D., Baldassarre M.E., Borrelli O., Thapar N., Vandenplas Y., Staiano A. (2019). Low FODMAPs diet for functional abdominal pain disorders in children: Critical review of current knowledge. J. Pediatr..

[81] Boradyn K.M., Przybylowicz K.E., Jarocka-Cyrta E. (2020). Low.FODMAP diet is not effective in children with functional abdominal pain: A randomized controlled trial. Ann. Nutr. Metab..

[82] Nurko S., Benninga M.A., Solari T., Chumpitazi B.P. (2022). Pediatric Aspects of Nutrition Interventions for Disorders of Gut-Brain Interaction. Am. J. Gastroenterol..

[83] Morreale C., Bresesti I., Bosi A., Baj A., Giaroni C., Agosti M., Salvatore S. (2022). Microbiota and Pain: Save Your Gut Feeling. Cells.

[84] Hambidge K.M. (2010). Micronutrient bioavailability: Dietary Reference Intakes and a future perspective. Am. J. Clin. Nutr..

